# Extracting T–S Fuzzy Models Using the Cuckoo Search Algorithm

**DOI:** 10.1155/2017/8942394

**Published:** 2017-07-06

**Authors:** Mourad Turki, Anis Sakly

**Affiliations:** Research Unit of Industrial Systems Study and Renewable Energy (ESIER), National Engineering School of Monastir (ENIM), 5019 Monastir, Tunisia

## Abstract

A new method called cuckoo search (CS) is used to extract and learn the Takagi–Sugeno (T–S) fuzzy model. In the proposed method, the particle or cuckoo of CS is formed by the structure of rules in terms of number and selected rules, the antecedent, and consequent parameters of the T–S fuzzy model. These parameters are learned simultaneously. The optimized T–S fuzzy model is validated by using three examples: the first a nonlinear plant modelling problem, the second a Box–Jenkins nonlinear system identification problem, and the third identification of nonlinear system, comparing the obtained results with other existing results of other methods. The proposed CS method gives an optimal T–S fuzzy model with fewer numbers of rules.

## 1. Introduction

To control any system, it is necessary to obtain an exact model of it but in many cases, it has not enough information to get an acceptable mathematical model, and it is required to use modelling techniques based on input–output data.

Fuzzy models are used due to their excellent performance in the modelling of nonlinear systems and being easy to implement. A fuzzy model is constructed from a basis of rules formed by inputs and outputs of a system [1].

The Takagi–Sugeno (T–S) fuzzy model is a type of fuzzy models which is able to give a local linear representation of the nonlinear system. Such a model is able to approximate a wide class of nonlinear systems because they are considered powerful in modelling and control of complex dynamic systems.

A T–S fuzzy model is powerful if it allows obtaining highly accurate models from a small number of rules but the majority of works in literature provide a large number of rules.

Many works concerning the T–S fuzzy models especially discrete-time type are done in literature such as in [2, 3]. The optimization of T–S fuzzy models is to determine the structure and parameters of model. The methods used to tune the antecedent and consequent parameters are the clustering algorithms [4] and the linear least squares [5–7]. The design of a fuzzy model is a search problem where each point represents a possible fuzzy model with different structures and parameters [1, 8]. In the aim to obtain optimal fuzzy models, many evolutionary algorithms (EAs) such as genetic algorithms (GAs) [9], genetic programming [10], evolutionary programming [11], evolution strategy [12], and differential evolution (DE) [13] have been used [14]. These methods tune the parameters and the structure is predefined.

Though, all parameters of model such as structure and parameters are linked and should be optimized simultaneously. Thereby, in [1] the authors have presented the optimization of rule structure where all the information is encoded into a chromosome.

The particle swarm optimization (PSO) is a novel metaheuristic algorithm used recently in many domains [15]. The PSO algorithm is used to elicit fuzzy models such as in [16] in which PSO optimize the structure, the number of membership functions, and the singleton consequent parameters. In [17], the results of PSO and GA are compared for the same method given in [16] with fixed number of rules and membership function for the same example of simulation.

The structure of the fuzzy model is identified using an online fuzzy clustering method and the parameters are optimized by PSO [20]. In [21], the fuzzy model is extracted using PSO with the recursive least-squares method. The ant colony and PSO were used to obtain T–S fuzzy model in [22]. Lin [23] used immune-based symbiotic PSO to obtain T–S models for the prediction of the skin color detection. In [24], Niu et al. used multiswarm PSO to tune fuzzy systems parameters. In [25], the subtractive clustering is utilized to extract a set of fuzzy rules and a variant of PSO called CPSO algorithm in the aim to find the optimal membership functions and the consequent parameters. In [19], the CRPSO is employed to tune all parameters of the fuzzy models. In [26], the GA is used for learning the T–S fuzzy model from data with a new encoding scheme.

DE and quantum inquired differential evolution is utilized to learn the T–S fuzzy model in [27]. T–S fuzzy models are also developed for modelling industrial systems such as the moving grate biomass furnace in [28].

Other metaheuristic named hunting search (HUS) is used in [18] to determine the parameters of the T–S fuzzy model.

A recent metaheuristic algorithm named the cuckoo search (CS) is proposed in 2009 by Yang. The CS algorithm has given best results compared to other metaheuristics such as PSO and GA. The CS is used to learn neural networks [29] and in the reliability optimization [30]. In [31], the CS is used to tune parameters of Sugeno-Type Fuzzy Logic Controller for liquid level control. Optimizing fuzzy controller using CS in the case study of computer numerical control of a steam condenser is done in [32]. In [33], a prediction of academic performance of student based on the CS is proposed. In [34, 35], the cuckoo search is used in the reduction of high order. In [36], Hammerstein model trained by the cuckoo search algorithm is proposed to identify nonlinear system. In [37], CS is used in the structural damage identification.

The goal and the motivation of this contribution is to obtain a T–S model with minimum number of rules by using a CS method. The objective is to have a model with less complexity able to be easily implemented in embedded systems and with a minimum of errors which proves its efficiency and precision.

This paper describes the use of CS to obtain the optimal structure in terms of a number of rules and also the parameters premises and consequents of T–S model simultaneously in the aim to explore the advantages of CS in optimization which are better compared to other metaheuristics in many examples in literature. The optimal T–S fuzzy models extracted are compared on the same examples with other methods in terms of MSE and number of rules.

This paper is organized as follows.  Section 2 describes the structure of T–S fuzzy system. The CS algorithm is introduced in Section 3.  Section 4 explains the encoding scheme of T–S model method used.  Section 5 presents the different metaheuristic algorithms.  Section 6 presents the application examples with results and discussions. Finally, conclusions are given in Section 7.

## 2. T–S Fuzzy Model

T–S fuzzy model presented in [38] is given by the following basis of rules.

*Rule i*. If *x*_1_ is *A*_*i*_^1^ and … and *x*_*N*_*I*__ is *A*_*i*_^*N*_*I*_^, then

(1) $$\begin{array}{c} y_{i}=\alpha_{i}^{0}+\alpha_{i}^{1}x_{1}+\cdots +\alpha_{i}^{N_{I}}x_{N_{I}}, \end{array}$$

where *i* = 1,…, *N*_*R*_, *N*_*R*_ is the number of rules, *x* = [*x*_1_,…, *x*_*N*_*I*__] represents the input, *N*_*I*_ is the size of input, *α*_*i*_^0^, *α*_*i*_^1^,…, *α*_*i*_^*N*_*I*_^ are the consequent parameters, *y*_*i*_ is the *i*th fuzzy rule output, and *A*_*i*_^*j*^ is a fuzzy subset.

The output of the model* y* is obtained as follows:

(2) $$\begin{array}{c} \hat{y}=\frac{\sum_{i=1}^{N_{R}}\omega_{i}y_{i}}{\sum_{i=1}^{N_{R}}\omega_{i}}, \end{array}$$

where *ω*_*i*_ of the *i*th rule is computed as

(3) $$\begin{array}{c} \omega_{i}=\overset{n}{\underset{j=1}{\prod }}\mu \left(\;A_{i}^{j}\;\right) \end{array}$$

with *μ*(*A*_*i*_^*j*^) being the membership function's grade of *A*_*i*_^*j*^ and is characterized by a Gaussian function as

(4) $$\begin{array}{c} \mu_{A_{i}^{j}}\left(\;x_{j}\;\right)=\exp\left(\;-\frac{1}{2}{\left(\;\frac{x_{j}-c_{i}^{j}}{\sigma_{i}^{j}}\;\right)}^{2}\;\right). \end{array}$$

*c*_*i*_^*j*^ and *σ*_*i*_^*j*^ are, respectively, the mean and the deviation of the MF. The premise parameters *c*_*i*_^*j*^ and *σ*_*i*_^*j*^ are adjusted [20].

## 3. Cuckoo Search

CS is developed by Yang and Deb in 2009 [39–41]. CS imitates the parasitism of cuckoo and used Lévy Flights which are better than random walks [32].

The CS has the specificity that the cuckoos lay their eggs in the nests of host birds. Some cuckoos can mimic the properties of the host eggs.

As a result, the number of the eggs abandoned is reduced and their reproductivity increases [42].

The CS models are used in many optimization problems. In [40, 43], it is demonstrated that Lévy Flights are better than random walk in CS algorithm.

In CS algorithm, the solution is given by an egg in a nest, and a new solution is represented by a cuckoo egg. The goal is to use the newer cuckoos or solutions to substitute worst solutions. In the classical algorithm, each nest has one egg; however the algorithm can be extended to complex problem [40, 43].

In the cuckoo search, the rules are as follows:

- Every cuckoo lays a single egg and throws it in a random nest.
- The best nests or solutions will be transferred to the next generations.
- The number of host nests is predefined, and a host can detect a stranger egg with probability *p*_*a*_ ∈ [0,1]. In that event, the host bird skips the egg or abandons the nest and builds a new nest in another place [43].

The CS algorithm is described in Pseudocode 1 [43].

In our work, *f*(*x*) is the MSE and nests *x*_*i*_ are possible T–S fuzzy models with cuckoos being the parameters of the T–S fuzzy model.

This algorithm uses a combination of a local random walk and the global random walk, controlled by parameter *p*_*a*_. The local random walk can be written as

(5) $$\begin{array}{c} x_{i}^{t+1}=x_{i}^{t}+\alpha s⊗H\left(\;p_{a}-\varepsilon \;\right)⊗\left(\;x_{j}^{t}-x_{k}^{t}\;\right), \end{array}$$

where *x*_*j*_^*t*^ and *x*_*k*_^*t*^ are two different solutions, *H*(*u*) is a Heaviside function, *ε* is a random number drawn from a uniform distribution, and *s* is the step size. The global random walk is carried out by using Lévy Flights.

(6) Hu=0,if  u<01,if  u≥0xit+1=xit+αLs,λ,

where

(7) $$\begin{array}{c} L\left(\;s,\lambda \;\right)=\frac{\lambda \Gamma \left(\lambda \right)\sin\left(\pi \lambda /2\right)}{\pi }\cdot \frac{1}{s^{1+\lambda }} \end{array}$$

with

(8) $$\begin{array}{c} \Gamma \left(\;\lambda \;\right)=\overset{+\infty }{\underset{0}{\int }}t^{\lambda -1}e^{-t}\;dt. \end{array}$$

Here, *α* > 0 is the step size scaling factor, which should be related to the scales of the problem of interest [44]. In fact, we use the Lévy Flights to obtain other T–S fuzzy models in the next generation which can be a solution.

Recent studies utilize cuckoo search such as Walton et al. by formulating a modified cuckoo search algorithm [45]; Yang and Deb improve it to multiobjective optimization [39].

## 4. Encoding Scheme for T–S Fuzzy Model

**Figure 1:**
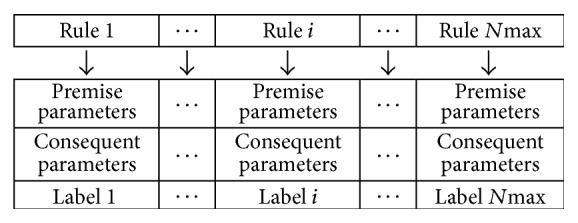
The particle structure of encoding a fuzzy rule base.

In this paper, the fuzzy system is given by a particle formed by the premise and the consequent parameters and also the labels which are used to choose the rules to construct the fuzzy system [20]. The fuzzy model's particle is presented in Figure 1.

In Figure 1, each rule *i* is formed by premise and consequent parameters and the label.

**Figure 2:**
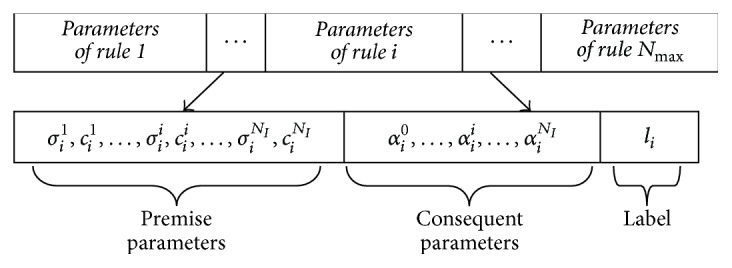
The encoding scheme of a fuzzy model.

Figure 2 shows that the particle *i* is given by a vector composed of the premise parameters *σ*_*i*_^1^, *c*_*i*_^1^,…, *σ*_*i*_^*N*_*I*_^, *c*_*i*_^*N*_*I*_^, consequent parameters *α*_*i*_^0^, *α*_*i*_^1^,…, *α*_*i*_^*N*_*I*_^, and the label *l*_*i*_ of all rules.

The fuzzy rules are selected using the labels in fact. If  *l*_*i*_ > 0, then the rule *i* is selected where *i* = 1,…, *N*_max_ is the index of the rule. The T–S fuzzy system is composed of the active rules.

The CS algorithm is used to elicit T–S fuzzy model and presented as follows:

(1) Encoding all the parameters premise and consequent of all rules with a predefined maximum number of rules *N*_max_.

(2) Defining a fitness function and the bounds of parameters.

(3) Randomizing an initial swarm of nests. Initializing all the parameters of particles representing fuzzy models with the lower and upper bounds chosen of parameters. Every nest is a fuzzy model with different structures and parameters.

(4) Calculating the fitness of initials nests which is MSE given by this equation:

(9) $$\begin{array}{c} MSE=\frac{1}{n}\overset{n}{\underset{k=1}{\sum }}{\left(\;y_{ref}\left(\;k\;\right)-y\left(\;k\;\right)\;\right)}^{2}. \end{array}$$

*n* is the number of input prototypes, *y*_ref_(*k*) is the desired output, and *y*(*k*) is the model output.

(5) Using the CS to search the optimal T–S fuzzy model.

**Step 1 .**

Get a fuzzy model (cuckoo) FM_1_ from the swarm of nests (TS fuzzy models generated randomly) randomly by using Lévy Flights, calculate its fitness, and select another fuzzy model (nest) FM_2_ randomly among the* n* nests of the swarm.

**Step 2 .**

If MSE(FM_1_) > MSE(FM_2_), replace FM_2_ by the new solution FM_1_; otherwise pass to the next step.

**Step 3 .**

*p*_*a*_ worst fuzzy models or nests (each nest is a fuzzy model in our work) are abandoned and new ones are built.

**Step 4 .**

Test the stopping criterion; if it is verified, keep the best fuzzy model (the optimal premise, consequent, and number of rules) and otherwise return to Step 1.

## 5. Metaheuristic Algorithms

There are many metaheuristic algorithms in literature such as the particle swarm optimization (PSO), the cooperative random learning PSO called CRPSO, the hunting search (HUS), the genetic algorithms (GA), and the differential evolution (DE).

### 5.1. The Particle Swarm Optimization (PSO)

The particle swarm optimization (PSO) imitates the movement of birds flocking or fish schooling looking for food [19]. The research of optimal solution is given by two equations:

(10) vt+1=w·vt+c1r1p−xt+c2r2pg−xtxt+1=xt+vt+1,

where *x* is the position of a particle, *v* is the velocity, *w* is the inertia weight, *c*_1_ and *c*_2_ are constants, *r*_1_ and *r*_2_ are random numbers between 0 and 1,* p* is the best position of the particle, and *p*_*g*_ is the best position of all particles in the swarm.

Another version of PSO is the cooperative random learning PSO (CRPSO) which used subswarms and the equation of velocity is given as follows:

(11) vjt+1=w·vjt+c1r1pj−xjt+c2r2pgj−xjt+c3r3pgr−xjt,

where *c*_3_ is constant, *r*_3_ is random number,* j* is the index of subswarm,* r* is the number between 1 and the number of subswarms, and *p*_*g*_ is the global best position of all subswarms [19].

### 5.2. The Hunting Search (HUS)

The hunting search (HUS) algorithm imitates the social behavior of animals when catching a prey in the way wolves hunt. The algorithm is based on approaching the leader having the best position in the group and reorganizing if the hunters are close to each other but still cannot find the optimum solution [18].

The research of new solution obeys this equation:

(12) $$\begin{array}{c} x_{i}=x_{i}+r\cdot NML\cdot \left(\;x_{i}^{l}-x_{i}\;\right), \end{array}$$

where *r* is a random number between 0 and 1, NML is the maximum number of movements toward the leader, and *x*_*i*_^*l*^ is the position of leader in the *i*th variable.

Another step is the reorganization of hunters which is given by this equation:

(13) xi=xil±rand·max⁡xi−min⁡xi·α·exp⁡−β·EN,

where EN is the number of past reorganizations and *α* and *β* are positive constants, with the aim to avoid falling into a local minimum and obtain a globally optimal solution.

### 5.3. The Genetic Algorithm (GA)

The genetic algorithm (GA) is a random search technique which imitates natural evolution with Darwinian survival of the fittest approach. In this algorithm, the variables are represented as genes in a chromosome, and the chromosomes are evaluated according to their fitness values. The chromosomes with better fitness are found through the three basic operations of GA: selection, crossover, and mutation [46].

The algorithm of GA is described as follows:

1. Initialization of initial population called chromosomes.
2. Evaluation of each element in the population by calculating its fitness function.
3. Selection of the chromosomes.
4. Generation of new chromosomes using the chromosomes selected and the GA's operations such as crossover and mutation.
5. Test of the stopping criterion: if validated, then the parameters are kept; otherwise return to Step (2).

### 5.4. The Differential Evolution (DE)

The differential evolution (DE) is a search algorithm that is similar to GA; it deals with a real coded population and devises its own crossover and mutation in the real space [13]. DE creates *x*^0^, a mutated form of any individual *x*, using the vector difference of randomly picked individuals called *x*^*∗*^ and *x*° using this equation:

(14) $$\begin{array}{c} x^{0}=x+\gamma \left(\;x^{*}-x^{{}^{\circ}}\;\right), \end{array}$$

where *γ* is a scaling factor between 0 and 2. Then, the crossover is applied between any individual member of the population and the mutated vector *x*^0^ and the best element is kept in the last iteration.

## 6. Application Examples and Discussions

In this part, the T–S models optimized are used to identify three systems: a nonlinear plant modelling problem, the Box–Jenkins gas furnace benchmark, and identification of nonlinear system.

The performance of CS is compared with other metaheuristic algorithms. The parameters used in the examples are presented in Table 1.

### 6.1. Application to Nonlinear Plant Modelling Problem

In [1, 5, 6], the nonlinear dynamic plant given by this nonlinear difference equation has been modelled.

(15) $$\begin{array}{c} y\left(\;k\;\right)=g\left(\;y\left(\;k-1\;\right),y\left(\;k-2\;\right)\;\right)+u\left(\;k\;\right), \end{array}$$

where

(16) gyk−1,yk−2=yk−1yk−2yk−1−0.51+yk−12+yk−22.

The aim of this application is to identify the nonlinear component *g*(*y*(*k* − 1), *y*(*k* − 2)) using the presented fuzzy model with CS algorithm. The example has two inputs and one output. The simulated data are formed by 400 points which are generated from the plant model: 200 data points are calculated using a random input signal *u*(*k*) uniformly distributed in [−1.5,1.5], and other 200 data points are computed using a sinusoidal input signal *u*(*k*) = sin⁡(2*π*k/25) [1, 5, 6].

The number of generations chosen is 500 and was iterated 50 times on a Pentium Core 2 Duo (1.8 GHz CPU) and 2 GB memory personal computer in the same computing environment (MATLAB 2007a). The consequent parameters are chosen in [−10,10] and the width of the premises parameters is given in [0,5].

As those in [1, 5, 6], we select *y*(*k* − 1) and *y*(*k* − 2) as inputs in the aim to predict the nonlinear component *g*(*y*(*k* − 1), *y*(*k* − 2)) and 5 is the maximum number of rules.  Table 2 gives the best results of 50 experimental trials.

According to Table 2, CS method gives the best results in terms of a number of rules (mean) fewer than the HUS method in [18], MSE (mean), and standard deviation (Std) in both training and testing stages compared with other methods. Also, the CS method gives good performances with the smaller number of evaluations than the result in [19]. In fact, in [19] 1000 generations and 20 particles are used with CRPSO algorithm and 2000 generations and 30 particles with PSO, GA, and DE; however in our work we use 500 generations and 20 particles which are less than the previous algorithms.

The optimal fuzzy model gives an MSE 8*∗*10^−4^ in training and 4*∗*10^−4^ in testing.

**Figure 3:**
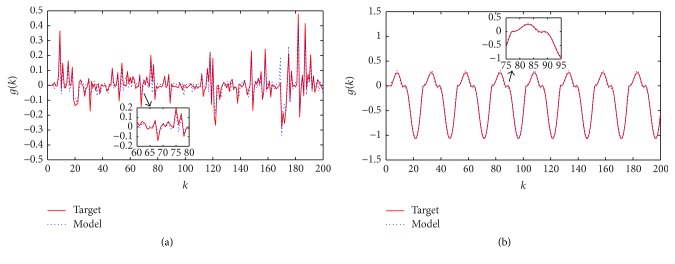
The target output and model output for nonlinear plant modelling problem: (a) training stage and (b) testing stage.

**Figure 4:**
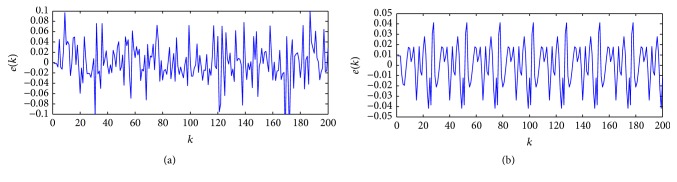
The errors between target output and model output: (a) training stage and (b) testing stage.

Figure 3 indicates outputs of target and model in the training and testing stages of the optimal model and the errors between them can be seen in Figure 4. As follows in Figure 3, the CS method gives the output with small errors.

### 6.2. Application to Box–Jenkins Gas Furnace Data

The Box–Jenkins gas furnace data [1, 11, 34–36] was recorded from a combustion process of a methane–air mixture [44]. The data set originally consists of 296 data points [*y*(*t*), *u*(*t*)]. The input *u*(*t*) is the gas flow rate; however the output *y*(*t*) was the carbon dioxide (CO_2_) concentration. The aim is to elicit a model to predict *y*(*t*) using this data. The first step is to determine the appropriate inputs to be used. The initial fuzzy inputs are *y*(*t* − 1), *y*(*t* − 2), *y*(*t* − 3), *y*(*t* − 4), *u*(*t* − 1), *u*(*t* − 2), *u*(*t* − 3), *u*(*t* − 4), *u*(*t* − 5), and *u*(*t* − 6), and the output is *y*(*t*). Due to many studies in literature, *y*(*t* − 1) and *u*(*t* − 4) are chosen as inputs. All the coefficients of the consequent parameters are chosen in [−100,100] and the width of the Gaussian function is limited to [0,10].

All the simulations are executed 50 times. The mean number of rules, the mean, and standard deviations of the MSE are listed in Table 3.

From Table 3, we conclude that CS has minimum mean MSE compared to PSO, HUS, and DE and less standard deviation MSE than PSO, CRPSO, HUS, and DE. The mean number of the rules of CS is much smaller than HUS, PSO, CPSO, GA, and DE. In conclusion, the CS-based method can give a fuzzy model with less number of rules. The optimal fuzzy model found by CS during 50 runs has an MSE of 0.139 and 3 rules.

**Figure 5:**
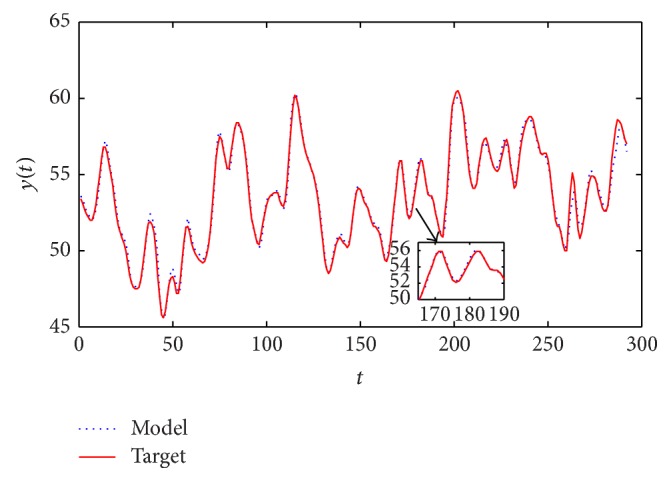
Fuzzy model output and real output for modelling the BJ gas furnace data.

**Figure 6:**
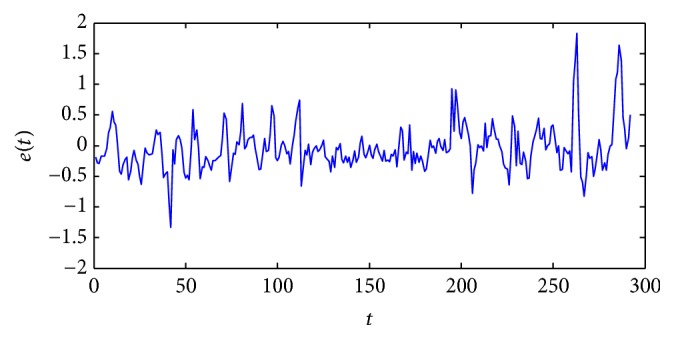
The errors between fuzzy model output and real output.

Figure 5 shows the target and the model outputs and Figure 6 gives the errors between them. The optimal fuzzy model can identify the output with small errors.

Table 4 gives the parameters of the optimal fuzzy model.

### 6.3. Identification of Nonlinear System

The third example used for identification, given by Narendra and Parthasarathy, is described by the next difference equation [47]:

(17) $$\begin{array}{c} y\left(\;k+1\;\right)=\frac{y\left(k\right)}{1+y^{2}\left(k\right)}+u^{3}\left(\;k\;\right). \end{array}$$

The input *u*(*k*) is given by this equation:

(18) $$\begin{array}{c} u\left(\;k\;\right)=\sin\left(\;\frac{2\pi k}{25}\;\right). \end{array}$$

The output of this equation depends nonlinearly on both its past values and the input. The 200 training input patterns are randomly generated in the interval $\left[\begin{array}{cc} -1 & 1 \end{array}\right]$ by using (17).

The aim of this application is to predict *y*(*k*) by using this approach when the inputs chosen are *y*(*k* − 1) and *u*(*k* − 1).

All the coefficients of the premise and consequent parameters are limited to [−5,5]. The maximum number of rules is chosen as 5.  Table 5 gives the best results of 50 experimental trials.

For all methods, the number of generations is fixed to 500 and the number of particles is fixed to 20.

As shown in Table 5, CS gives a minimum number of rules, less mean of MSE, and less standard deviation MSE compared to PSO, HUS, and GA.

The optimal fuzzy model found by CS during 50 runs has an MSE of 0.0231 and 3 rules.

**Figure 7:**
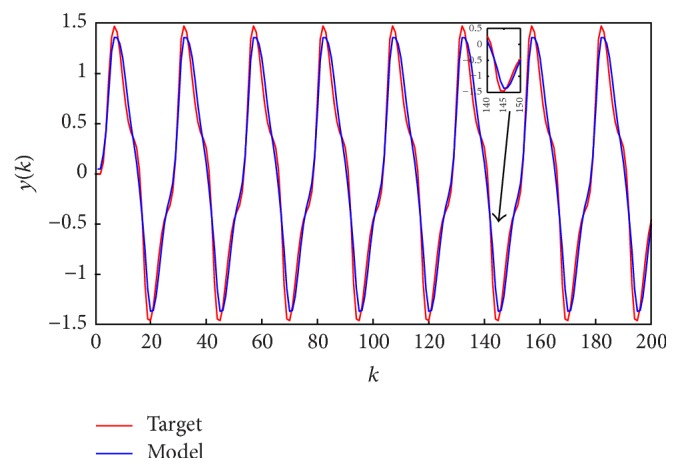
Fuzzy model output and target output for identification of the nonlinear system.

**Figure 8:**
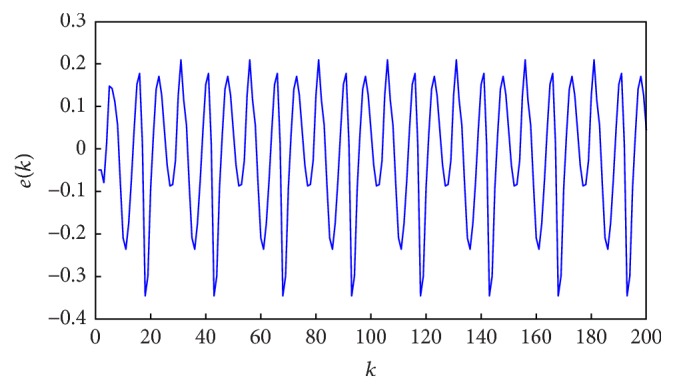
The errors between fuzzy model output and target output.

Figure 7 shows the target and the model outputs and Figure 8 gives the errors between them. The optimal fuzzy model can predict the output with small errors.

## 7. Conclusions

In this paper, the extracting of T–S fuzzy model using CS algorithm is described. The T–S fuzzy model tuned by CS has the rules structures and both the premises and consequents parameters optimized. The optimal T–S fuzzy model has a fewer number of rules and smaller MSE both in mean and in standard deviation. The T–S model using CS algorithm is validated by the comparison of its performance to other methods for modelling three benchmarks: the nonlinear plant modelling problem, the Box–Jenkins problem, and identification of nonlinear system and this shows that the CS algorithm gives much better accuracy in modelling nonlinear systems; in fact, CS gives a model with minimum of number of rules with better errors compared to other metaheuristics.

## Figures and Tables

**Pseudocode 1:**
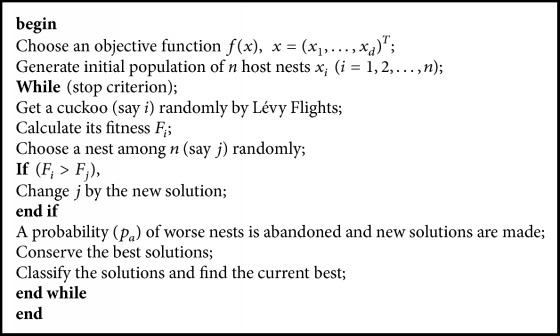


**Table 1:** CS parameter values.

Parameter	Value
Number of nests	20
*p*_*a*_	0.25
Number of iterations	500
*λ*	1.5
Step size *α*	1
Maximum number of rules *N*max	5

**Table 2:** Performance comparison of different methods for nonlinear plant modelling.

Method	Number of rules	MSE (training)		MSE (testing)	
	Mean	Mean	Std	Mean	Std
*CS*	*3.3*	*0.0016*	*0.0005*	*0.0009*	*0.0003*
HUS [18]	4.84	0.0017	0.0015	0.0029	0.0016
CRPSO [19]	5.6	0.0020	0.0016	0.0035	0.0044
PSO [19]	5.64	0.0055	0.0055	0.0063	0.0072
GA [19]	3.62	0.0038	0.0053	0.0045	0.0051
DE [19]	5.32	0.0081	0.0041	0.0086	0.0062

**Table 3:** Performance comparison of different methods for Box–Jenkins gas furnace data set.

Method	Number of rules (mean)	MSE (mean)	MSE (Std)
*CS *	*2.34*	*0.1527*	*0.0129*
HUS	2.68	0.1796	0.0300
PSO [19]	3.64	0.1550	0.0427
CRPSO [19]	3.96	0.1428	0.0138
GA [19]	4.32	0.1474	0.0109
DE [19]	4.96	0.1768	0.0602

**Table 4:** The optimal fuzzy model for identification of Box–Jenkins gas furnace problem.

Rule base	*y*(*t* − 1)(*σ*_1_, *c*_1_)	*u*(*t* − 4)(*σ*_2_, *c*_2_)	*y*(*t*)
Rule 1	(4.64,48.36)	(2.72,2.00)	−0.32*y*(*t* − 1) − 2.67*u*(*t* − 4) + 72.61
Rule 2	(4.77,50.72)	(1.86,2.49)	1.91*y*(*t* − 1) + 1.23*u*(*t* − 4) − 51.66
Rule 3	(2.67,48.00)	(1.32, −1.91)	2.20*y*(*t* − 1) + 3.67*u*(*t* − 4) − 62.00

**Table 5:** Performance comparison of different methods for identification of nonlinear system.

Method	Number of rules (mean)	MSE (mean)	MSE (Std)
*CS *	*2.40*	*0.0263*	*0.0031*
HUS	3.08	0.0192	0.0116
PSO	2.54	0.0330	0.0059
GA	2.64	0.1236	0.1694

## References

[1] Kim M.-S., Kim C.-H., Lee J.-J. (2006). Evolving compact and interpretable Takagi-Sugeno fuzzy models with a new encoding scheme. *IEEE Transactions on Systems, Man, and Cybernetics, Part B: Cybernetics*.

[2] Xie X., Yue D., Ma T., Zhu X. (2014). Further studies on control synthesis of discrete-time T-S Fuzzy systems via augmented multi-indexed matrix approach. *IEEE Transactions on Cybernetics*.

[3] Xie X., Yue D., Zhang H., Xue Y. (2017). Fault Estimation Observer Design for Discrete-Time Takagi-Sugeno Fuzzy Systems Based on Homogenous Polynomially Parameter-Dependent Lyapunov Functions. *IEEE Transactions on Cybernetics*.

[4] Singh K. K., Nigam M. J., Pal K., Mehrotra A. (2014). A fuzzy Kohonen local information c-means clustering for remote sensing imagery. *IETE Technical Review (Institution of Electronics and Telecommunication Engineers, India)*.

[5] Rong H.-J., Sundararajan N., Huang G.-B., Saratchandran P. (2006). Sequential adaptive fuzzy inference system (SAFIS) for nonlinear system identification and prediction. *Fuzzy Sets and Systems*.

[6] Wang L., Yen J. (1999). Extracting fuzzy rules for system modeling using a hybrid of genetic algorithms and Kalman filter. *Fuzzy Sets and Systems. An International Journal in Information Science and Engineering*.

[7] Angelov P. P., Filev D. P. (2004). An approach to online identification of Takagi-Sugeno fuzzy models. *IEEE Transactions on Systems, Man, and Cybernetics, Part B: Cybernetics*.

[8] Shi Y., Eberhart R., Chen Y. (1999). Implementation of evolutionary fuzzy systems. *IEEE Transactions on Fuzzy Systems*.

[9] Cordón O., Herrera F. (1999). A two-stage evolutionary process for designing TSK fuzzy rule-based systems. *IEEE Transactions on Systems, Man, and Cybernetics, Part B: Cybernetics*.

[10] Cordon O., Herrera F., Gomide F., Hoffmann F., Magdalena L. Ten years of genetic fuzzy systems: current framework and new trends.

[11] Kang S.-J., Woo C.-H., Hwang H.-S., Woo K. B. (2000). Evolutionary design of fuzzy rule base for nonlinear system modeling and control. *IEEE Transactions on Fuzzy Systems*.

[12] Pedrycz W., Reformat M. (2003). Evolutionary fuzzy modeling. *IEEE Transactions on Fuzzy Systems*.

[13] Eftekhari M., Katebi S. D., Karimi M., Jahanmiri A. H. (2008). Eliciting transparent fuzzy model using differential evolution. *Applied Soft Computing Journal*.

[14] Mitra S., Hayashi Y. (2000). Neuro-fuzzy rule generation: survey in soft computing framework. *IEEE Transactions on Neural Networks*.

[15] Mangat V., Vig R. (2014). Dynamic PSO-based associative classifier for medical datasets. *IETE Technical Review (Institution of Electronics and Telecommunication Engineers, India)*.

[16] Khosla A., Kumar S., Aggarwal K. K. A framework for identification of fuzzy models through particle swarm optimization algorithm.

[17] Khosla A., Kumar S., Ghosh K. R. A comparison of computational efforts between particle swarm optimization and genetic algorithm for identification of fuzzy models.

[18] Bouzaida S., Sakly A., M'Sahli F. (2014). Extracting TSK-type neuro-fuzzy model using the hunting search algorithm. *International Journal of General Systems*.

[19] Zhao L., Qian F., Yang Y., Zeng Y., Su H. (2010). Automatically extracting T-S fuzzy models using cooperative random learning particle swarm optimization. *Applied Soft Computing Journal*.

[20] Juang C.-F., Chung I.-F., Hsu C.-H. (2007). Automatic construction of feedfoward/recurrent fuzzy systems by clustering-aided simplex particle swarm optimization. *Fuzzy Sets and Systems. An International Journal in Information Science and Engineering*.

[21] Chen C.-C. (2006). A PSO-based method for extracting fuzzy rules directly from numerical data. *Cybernetics and Systems*.

[22] Juang C.-F., Lo C. (2008). Zero-order {TSK}-type fuzzy system learning using a two-phase swarm intelligence algorithm. *Fuzzy Sets and Systems. An International Journal in Information Science and Engineering*.

[23] Lin C.-J. (2008). An efficient immune-based symbiotic particle swarm optimization learning algorithm for {TSK}-type neuro-fuzzy networks design. *Fuzzy Sets and Systems. An International Journal in Information Science and Engineering*.

[24] Niu B., Zhu Y., He X., Shen H. (2008). A multi-swarm optimizer based fuzzy modeling approach for dynamic systems processing. *Neurocomputing*.

[25] Zhao L., Yang Y., Zeng Y. (2009). Eliciting compact T-S fuzzy models using subtractive clustering and coevolutionary particle swarm optimization. *Neurocomputing*.

[26] Du H., Zhang N. (2008). Application of evolving Takagi-Sugeno fuzzy model to nonlinear system identification. *Applied Soft Computing Journal*.

[27] Su H., Yang Y. (2011). Differential evolution and quantum-inquired differential evolution for evolving Takagi-Sugeno fuzzy models. *Expert Systems with Applications*.

[28] Grosswindhager S., Haffner L., Voigt A., Kozek M. (2014). Fuzzy modelling of a moving grate biomass furnace for simulation and control purposes. *Mathematical and Computer Modelling of Dynamical Systems. Methods, Tools and Applications in Engineering and Related Sciences*.

[29] Valian E., Mohanna S., Tavakoli S. (2011). Improved Cuckoo Search Algorithm for Feed forward Neural Network Training. *International Journal of Artificial Intelligence & Applications*.

[30] Valian E., Tavakoli S., Mohanna S., Haghi A. (2013). Improved cuckoo search for reliability optimization problems. *Computers & Industrial Engineering*.

[31] Aghaei A., Kiani K., Bayati M. (December 2014). Optimizing fuzzy controller using cuckoo optimization algorithm (COA). *International Journal of Enhanced Research in Science Technology & Engineering*.

[32] Chen J. F., Do Q. H. (Sep 2014). A cooperative cuckoo search – hierarchical adaptive neuro-fuzzy inference system approach for predicting student academic performance. *Journal of Intelligent & Fuzzy Systems: Applications in Engineering and Technology*.

[33] Box G. E. P., Jenkins G. M. (1970). *Time Series Analysis, Forecasting and Control*.

[34] Afzal Sikander A., Rajendra Prasad B. (2015). A novel order reduction method using cuckoo search algorithm. *IETE Journal of Research*.

[35] Narwal A., Prasad B. R. (2016). A novel order reduction approach for LTI systems using cuckoo search optimization and stability equation. *IETE Journal of Research*.

[36] Gotmare A., Patidar R., George N. V. (2015). Nonlinear system identification using a cuckoo search optimized adaptive Hammerstein model. *Expert Systems with Applications*.

[37] Xu H. J., Liu J. K., Lu Z. R. (2016). Structural damage identification based on cuckoo search algorithm. *Advances in Structural Engineering*.

[38] Takagi T., Sugeno M. (1985). Fuzzy identification of systems and its applications to modeling and control. *IEEE Transactions on Systems, Man and Cybernetics*.

[39] Yang X.-S., Deb S. (2013). Multiobjective cuckoo search for design optimization. *Computers & Operations Research*.

[40] Yang X.-S., Deb S. (2010). Engineering optimisation by Cuckoo search. *International Journal of Mathematical Modelling and Numerical Optimisation*.

[41] Balochian S., Ebrahimi E. (2013). Parameter Optimization via Cuckoo Optimization Algorithm of Fuzzy Controller for Liquid Level Control. *Journal of Engineering (United States)*.

[42] Payne R., Sorenson M., Klitz K. (2005). *The Cuckoos*.

[43] Yang X.-S., Deb S. Cuckoo search via Lévy flights.

[44] Yang X., Deb S. (2014). Cuckoo search: recent advances and applications. *Neural Computing and Applications*.

[45] Walton S., Hassan O., Morgan K., Brown M. R. (2011). Modified cuckoo search: a new gradient free optimisation algorithm. *Chaos, Solitons and Fractals*.

[46] Turki M., Bouzaida S., Sakly A., M'Sahli F. (2014). Modeling and on line control of nonlinear systems using neuro-fuzzy learning tuned by metaheuristic algorithms. *International Journal of Control and Automation*.

[47] Narendra K. S., Parthasarathy K. (1990). Identification and control of dynamical systems using neural networks. *IEEE Transactions on Neural Networks*.

